# Multi-Instance Metric Transfer Learning for Genome-Wide Protein Function Prediction

**DOI:** 10.1038/srep41831

**Published:** 2017-02-06

**Authors:** Yonghui Xu, Huaqing Min, Qingyao Wu, Hengjie Song, Bicui Ye

**Affiliations:** 1 School of Computer Science and Engineering, South China University of Technology, Guangzhou, 510006, China; 2 School of Software Engineering, South China University of Technology, Guangzhou, 510006, China; 3 State Key Laboratory for Novel Software Technology, Nanjing University, China; 4 Wuzhou Red Cross Hospital, Wuzhou, 543002, China

## Abstract

Multi-Instance (MI) learning has been proven to be effective for the genome-wide protein function prediction problems where each training example is associated with multiple instances. Many studies in this literature attempted to find an appropriate Multi-Instance Learning (MIL) method for genome-wide protein function prediction under a usual assumption, the underlying distribution from testing data (target domain, *i*.*e*., TD) is the same as that from training data (source domain, *i*.*e*., SD). However, this assumption may be violated in real practice. To tackle this problem, in this paper, we propose a Multi-Instance Metric Transfer Learning (MIMTL) approach for genome-wide protein function prediction. In MIMTL, we first transfer the source domain distribution to the target domain distribution by utilizing the bag weights. Then, we construct a distance metric learning method with the reweighted bags. At last, we develop an alternative optimization scheme for MIMTL. Comprehensive experimental evidence on seven real-world organisms verifies the effectiveness and efficiency of the proposed MIMTL approach over several state-of-the-art methods.

During the past decades, a variety of computational methods have been proposed to tackle the genome-wide protein function prediction problem^1^,^2^,^3^. Some research in this literature^4^,^5^ attempt to solve the protein function prediction problem as a naturally and inherently multi-instance learning problem. Multi-Instance Learning^6^,^7^ is a recent machine learning framework for the learning problem in which each training example is represented by a bag of instances. In MIL, a bag has a positive label if it contains at least one positive instance. Otherwise the bag is annotated with a negative label.

MIL has received considerable attention and been frequently applied in a wide range of real-world applications^2^,^8^ since it is more convenient and natural for representing complicated objects which have multiple semantic meanings. With the help of the MIL, some inherent patterns which are closely related to some labels may become explicit and clearer. Based on the advantage of MI representation, a variety of MIL methods have been proposed. Conventional step of MIL considers the availability of a large amount of labeled training data to learn a classifier in a source domain, and predicts the label of the test data in the same domain. However, in many real-world applications, labeled data are limited and expensive to obtain. This is especially true for genome data. If we can transfer information from a similar domain to assist MIL, it will be helpful.

Transfer learning^9^,^10^,^11^,^12^,^13^,^14^,^15^ has been developed to handle the situation in which a classification task with sufficient training data is considered as source domain (SD), and we have limited data in another target domain (TD) where the latter data may be in a different feature space or have a different distribution. If we can encode the transfer learning methods into MIL, we can reuse the labeled data in the source domain to the target task. Recently, a number of transfer learning methods^16^,^17^ have been developed. Most of these transfer learning algorithms are designed for single-instance learning (SIL) where training example is represented by one instance. It has been shown that learning performance can be significantly enhanced if the transfer learning techniques is exploited in SIL. However, it is difficult to directly apply these transfer learning methods to multi-instance situation. Hence, it is urgent to develop a MIL method under transfer learning setting.

Furthermore, most of these transfer learning methods^16^,^17^ are based on the Euclidean distance idea, i.e., an objective function is optimized to maintain the class information of examples by their Euclidean distances. Unfortunately, objective functions based on the Euclidean distance are inappropriate to maximize the distance of the bags between classes, while minimizing that within each class for MI data^16^,^18^,^19^,^20^. This is because the Euclidean distance cannot exploit the intrinsic geometry among the data. To tackle this problem, it is urgent to develop a suitable distance metric for MIL method under transfer learning setting.

In this paper, we proposed a new multi-instance learning algorithm for transfer learning setting, called Multi-Instance Metric Transfer Learning (MIMTL). Compared with other state-of-the-art MIL algorithms, it is worthwhile to highlight the following two aspects of our proposed approach here:

- Different from many MIL approaches that only suitable for the traditional MIL problem, we propose a MIL approach under transfer learning setting. By utilizing the bag importance weights, we transfer knowledge from a source domain to target domain. Such that, we can reuse the SD bags to train a classifier for the target task in TD. By this way, our algorithm can significantly improve the prediction performance against the traditional MIL approaches.
- Compared to most MIL approaches which fit the data in a Euclidean space^1^,^2^,^3^, we exploit the intrinsic geometry of the MI data by using the Mahalanobis distance^21^,^22^,^23^,^24^. With the outstanding characteristic of the Mahalanobis distance (i.e., taking into account the correlations among different domains data, unit less and scale-invariant), our approach can be more applicable to respect the intrinsic geometric structure of the data from domains.

The rest of the paper is structured as follows. We briefly review the related works. We next introduce our proposed algorithm MIMTL and present an optional optimization scheme for the proposed algorithm. Then, we test the performance of our algorithm and present the experimental results on several benchmark data sets. Finally, we conclude this paper and present the future work.

## Related Works

Previous studies related to our work can be classified into three categories: *traditional MIL, metric learning based MIL* and *transfer learning based MIL*.

### Traditional MIL

Multi-Instance Multi-Label k-Nearest Neighbor (MIMLkNN)^25^ try to utilize the popular *k*-nearest neighbor techniques into MIL. Motivated by the advantage of the citers that is used in Citation-kNN approach^26^, MIMLkNN not only considers the test instances’ neighboring examples in the training set, but also considers those training examples which regard the test instance as their own neighbors (*i*.*e*., the citers). Different from MIMLkNN, Multi-instance Multi-label Support Vector Machine (MIMLSVM)^6^ first degenerates MIL task to a simplified single-instance learning (SIL) task by utilizing a clustering-based representation transformation^6^,^27^. After this transformation, each training bag is transformed into a single instance. By this way, MIMLSVM maps the MIL problem to a SIL problem. For another traditional MIL approach, Multi-instance Multi-label Neural Network (MIMLNN)^28^ is obtained by using the two-layer neural network structure^28^ to replace the Multi-Label Support Vector Machine (MLSVM)^6^ used in MIMLSVM.

### Metric based MIL

Different from MIMLNN, to encode much geometry information of the bag data, the metric-based Ensemble Multi-Instance Multi-Label (EnMIMLNN)^4^ combines three different Hausdorff distances (*i*.*e*., average, maximal and minimal) to define the distance between two bag, and proposes two voting-based models (*i*.*e*., EnMIMLNN*voting*_1_ and EnMIMLNN*voting*_2_). Recently, Xu Ye *et al*. proposes a metric-based multi-instance learning method (MIMEL)^29^ by minimizing the KL divergence between two multivariate Gaussians with the constraints of maximizing the distance of bags between class and minimizing the distance of bags within class. Different from MIMEL, Jin Rong *et al*. proposes a metric-based learning method^30^ for multi-instance multi-label problem. Recently, MIML-DML^5^ attempts to find a distance metric by considering that the same category bag pairs should have a smaller distance than that from different categories. These metric-based MIL approaches are both designed for the traditional MIL problem where the bags in SD and TD are drawn from the same distribution.

### Transfer learning based MIL

Recently, MICS^31^ proposed tackling the MI co-variate shift problem by considering the distribution change at both bag-level and instance-level. MICS attempts to utilize the weights of bags and instances to solve the covariate shift problem. Then, with the learned weights, the MI co-variate shift problem can be solved by traditional MIL methods. However, MICS does not present the method to utilize the learned weights into multi-instance metric learning.

## Method

In this section, we first give some definitions corresponding to MIL for genome-wide protein function prediction. Then, we present the traditional multi-instance metric learning approach briefly and discuss the limitation of the approach under transfer learning setting. After the discussion, a bag weights estimation method is presented. At last, we present the proposed method based on the learned bag weights.

The genome-wide protein function prediction problem aims to find a method to annotate the biological function for a given protein. However, the structure and function of proteins are very complex. In fact, many proteins often include several structural domains, and each domain may appear in a number of different proteins. These domains can be treated as distinct functional or structural units of a protein. Multi-domain proteins are likely to create new functions by emerging from a selective pressure during evolution. A wide variety of proteins have diverged from common ancestors by combining and associating different domains. Nature often gets together several domains to produce multi-domain and multifunctional proteins with a large number of possibilities^32^. To describe the complex structure of proteins, we utilize the MIL into the genome-wide protein function problem. For instance, we represent each domain with an input instance, and represent each protein in organisms with a bag of instances, and treat each biological function as an output label. Thus, the protein function prediction problem is naturally and inherently MIL learning tasks.

Formally, We denote by 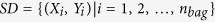 the training dataset, where 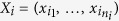 represent the *i*-th protein in the training set. *X*_*i*_ is a bag of *n*_*i*_ instances, and every instance 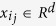 is a vector of *d* dimensions. *n*_*bag*_ indicates the bag number in *SD*. 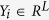 representes the Gene Ontology terms^4^ assigned to *X*_*i*_. *Y*_*i*_ is a binary vector, and 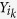 indicates the *k*-th element in *Y*_*i*_. 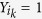 indicates that the *i*-th protein is associated with the *k*-th Gene Ontology term. In other words, the bag *X*_*i*_ is assigned to class *ϑ*_*k*_, and 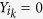 otherwise. We assume that bag *X*_*i*_ is assigned to *ϑ*_*k*_ at least one instance in *X*_*i*_ belongs to *ϑ*_*k*_. Formally, MIL task aims to learn a function 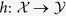 from the given data set SD. Important definitions are shown in Table 1.

### Multi-Instance Metric Learning

After the definition, we briefly formulate the multi-instance metric learning framework which is provided by the paper^5^. The learning framework aims to find a distance metric *M* = *A*^T^*A, M* ∈ *R*^*d*×*d*^, *A* ∈ *R*^*d×d*^ and a hypothesis 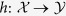 from the training data *SD*. In general, the multi-instance metric learning problem can be formulated as the following optimization problem:


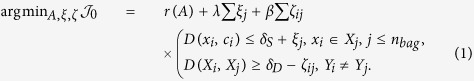


The first constraint in equation (1) is used to minimize the instance distance in each bag^5^. The second constraint in equation (1) is used to maximize the distance between bags that corresponding to different labels. r(A) is a regularization term for *A, λ* and *β* are two balance parameters. Some times the training data extracted from the proteins may contain some noise and the noise may reduce the performance of the algorithm. To solve this problem, two slack vectors *ξ* and *ζ* are utilized into the learning framework to improve the robustness of the algorithm. *δ*_*S*_ and *δ*_*D*_ are two constants (*δ*_*S*_ < *δ*_*D*_). *δ*_*S*_ is used to limit the maximum distance between the center of the bag and the instance in the bag. *δ*_*D*_ is used to limit the minimum distance between bags from different class. *c*_*i*_ is the center of *X*_*i*_. *D*(*x*_*i*_, *c*_*i*_) is the square of the Mahalanobis distance between instance *x*_*i*_ and *c*_*i*_ and *D*(*X*_*i*_, *X*_*j*_) is the square of the Mahalanobis distance between bags *X*_*i*_ and *X*_*j*_. Here, we can define *D*(*x*_*i*_, *c*_*i*_) = (*x*_*i*_ − *c*_*i*_)^T^ *A*^T^ *A*(*x*_*i*_ − *c*_*i*_) and *D*(*X*_*i*_, *X*_*j*_) as,


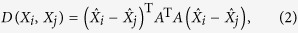


where 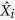 indicates the average of all the instances in bag *X*_*i*_. According to this method, we can get together the instances from the same protein and separate the proteins with different biological function.

#### Loss Analysis of Multi-Instance Metric Learning

The learning framework presented in equation (1) is designed for the learning problem where training and test data are drawn from same distribution. However, in many real applications, this assumption cannot be guaranteed. For more specific analysis of the drawback of the learning framework, we first discuss the loss analysis of equation (1) under transfer learning setting in this section.

**Figure 1:**
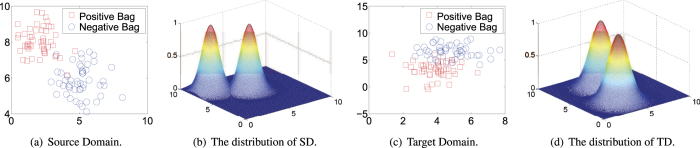
An example to show the different distributions of source domain and target domain.

Before we present the loss analysis, we first use an example to more intuitively explain the different distributions between training and test data. Figure 1 gives an example in which the distributions of the source domain and target domain are different. In such case, the distance metric learned by equation (1) can not help to minimize the distance in each bag and maximize the distance between bags from different classes due to the fact that, the expected loss of equation (1) on SD is inconsistent with that on TD. Herein, we define the expected loss corresponding to equation (1) on SD as following,


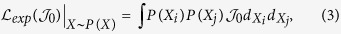


where *P*(*X*) indicates the density of bag *X* in SD. Equation (3) can be refined as,


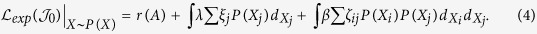


Similarly, the expected loss corresponding to equation (1) on TD can be written as,


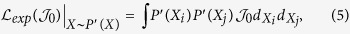


*P*′(*X*) represents the density of bag *X* in TD. From equations (3) and (5), we can find that if 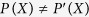, the expected loss on SD is not equal to that on TD,


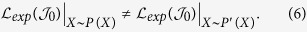


In other words, if the training bags number of SD is as large as possible (*i*.*e*., to be the infinity), equation (1) still can not generate an optimal solution for the multi-instance prediction problem in TD.

### Bag Weights Estimation

After the loss analysis of Multi-Instance Metric Learning, we find that the traditional learning framework in equation (1) is not suitable for the learning problem under transfer learning setting. The main reason for this situation is the divergence between the expected loss of equation (1) on SD and TD. In this section, we propose learning a suitable bag weights to solve this problem.

From equations (3), (5) and (6), it is obvious that, if there exist a suitable bag weight for each bag *X* in SD to satisfy the following equation,


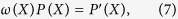


we can balance the difference between 
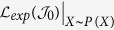
 and 
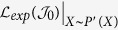
. To estimate the bag weights *ω*(*X*), we set *ω*(*X*) = *P*′(*X*)/*P*(*X*) and adopt the approach proposed by MICS^31^ where the bag weights is considered as a function that can be approximated by a linear combination of some basic functions, i.e., 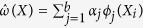, where {*ϕ*_*j*_}’s indicate a set of pre-defined basis functions and {*α*_*j*_}’s represent the corresponding nonnegative parameters to be learned. The weights of source-domain bags, *ω*(*X*), can be obtained by minimizing the least square loss 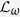 between *ω* and 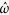, i.e.,


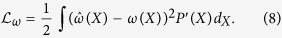


As shown in MICS^31^, equation (8) can be converted to the following optimization problem,


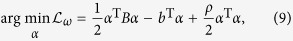


where *B* is a *p* × *p* matrix and *b* is a *p* × 1 vector,


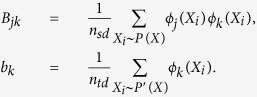


The basis functions *ψ*(*X*) can be selected as a series of kernels. Followed by the work of MICS^31^, we use the MI-Kernel^33^ to measure the similar or dissimilar between multi-instance bags,


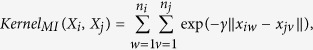


where *γ* is the kernel width.

Since the optimization problem equation (9) is convex, gradient ascent approaches can be applied to obtain the global solution. By this way, we can learn a weight for each bag. According to the learned bags we can balance the differnece between 
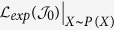
 and 
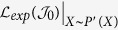
.

### Multi-Instance Metric Transfer Learning

We have balanced the divergence between the expected loss of equation (1) on SD and TD by reweighting the training bags. However, we still do not know how to obtain a Mahalanobis distance under the transfer learning setting. In this section, we will utilize the learned bag weight vector into our learning framework to obtain a Mahalanobis distance metric under transfer learning setting.

Based on the loss analysis of multi-instance metric learning and the learned bag weights, we can reformulate equation (1) as follows,


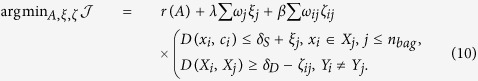


Note that a preprocessing step to centralize the input data is performed in MIMTL,


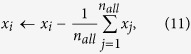


where *n*_*all*_ indicates all the instances number in *SD*, then *D*(*x*_*i*_, *c*_*i*_) can be represented as,


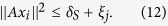


In the following, without special declaration the data are supposed to be centralized.

For some situations, in addition to the bags from SD, we can obtain few labeled bags from TD. Hence, we can learn the distance metric *A* based on the labeled bags both in SD and TD by setting the weights of bags from TD to be 1. Then we obtain the optimization problem,


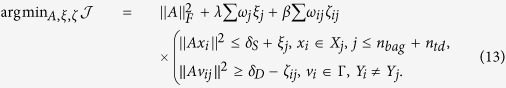


where 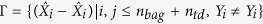, *n*_*td*_ is the labeled bag’s number in TD. Note that, if we cannot obtain any labeled bags from TD, we can delete the constraints in equation (13) corresponding to TD, and formulate the following method to learn the distance metric *A*,


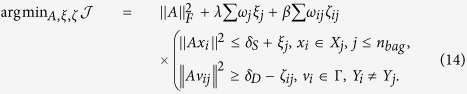


Since we have reweighted the bags to balance the expected loss of learning framework on SD and TD, equation (14) can also generate an optimal solution for the multi-instance prediction problem, even without labeled bags in TD. In other words, labeled bags from TD are not required to guarantee a consistent expected loss of equation (13) on SD and TD.

#### Loss Analysis of Multi-Instance Metric Transfer Learning

We have provided a new learning framework in equations (13) and (14) to learn Mahalanobis distance metric under transfer learning setting. For a more detailed understanding of the new learning framework, we analyze the expected loss of this framework on both SD and TD in this section.

We use 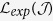 to represent the expected loss of equation (13). Then 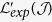 can be written as,


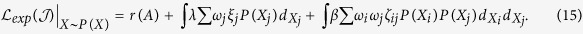


Because *ω*(*X*) = *P*′(*X*)/*P*(*X*), equation (15) can be rewritten as,


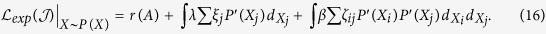


From equation (16), we can see that the expected loss of equation (13) on SD is consistent with that on TD,


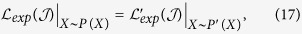


where 
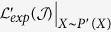
 indicates the expected loss of equation (13) on TD. This means that we can balance the difference between the expected loss of equation (13) on SD and TD with the help of the learned bag weights. Such that, equation (13) can guarantee the ability of generalizing the predicted model of TD data. Hence, distance metric *A* learned by MIMTL can more effectively measure the distance between bags.

### Prediction by Using the Learned Metric

After we obtain the Mahalanobis distance metric by solving the optimization problem in equation (13), we can predict the label for test bags in TD. In this section, we will present how to predict by using the learned distance metric.

After we obtain the distance metric *A*, a base multi-instance learner (*i*.*e*., the citation-kNN algorithm^26^ and the multi-instacne multi-label support vector machine^6^) can be used cooperating with the distance metric *A* for bag label predicting. Considering the fact that, most of the genome-wide protein function prediction problem are associated with multiple class labels^4^,^34^, we train an independent distance metric *A* and a base multi-instance learner for each class. We present two methods to cooperate the distance metric *A* with basic multi-instance learner as following.

#### The First Method for Prediction

For the first method, we use MIMTL_*kNN*_ to represent the MIMTL which use the citation-kNN algorithm as the base learner. For a given test bag *X*_*i*_ and the distance metric *A*, MIMTL_*kNN*_ compute the distance between *X*_*i*_ and each training bag. Then, we find both the references and citers of *X*_*i*_. The class labels of *X*_*i*_ is determined by a majority vote of the *r* nearest reference bags and the *c* nearest citing bags corresponding to *X*_*i*_.


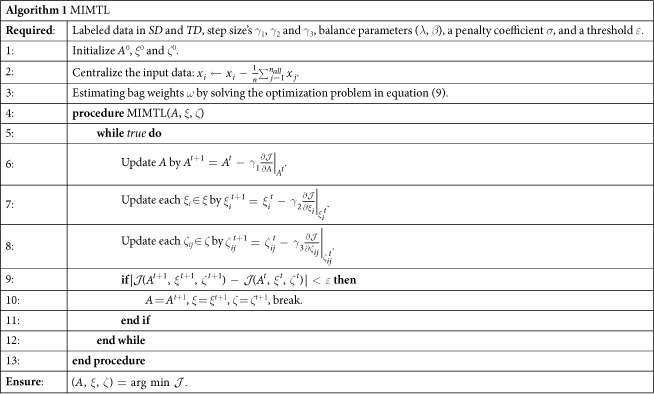


#### The Second Method for Prediction

For the second method, we use MIMTL_*SVM*_ to represent the MIMTL which utilize the MIMLSVM algorithm as the base learner. To predict the label of a given test bag *X*_*i*_, we first cluster all of the instances in training data into *k*-medoids with the learned distance metric *A*. We denote the cluster number 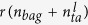, where *r* represents the ratio parameter and 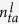 is the labeled bags number in TD (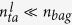). Then, we generate a *k*-dimensional numerical vector for each bag in SD and TD 
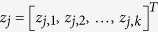
, 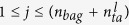, in which *z*_*j*,*i*_ denotes the distance between the *j*-th bag and the *i*-th medoid. Then we can transfer the bag data set into a single-instance data set *Z*,


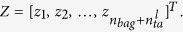


With derived data set *Z*, we learn a binary classifier by support vector machine^35^. For the given test bag *X*_*i*_, we first transform *X*_*i*_ into a *k*-dimensional numerical vector 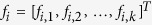 by computing the Mahalanobis distance between bag *X*_*i*_ and all the medoids. Then, we can predict the label of *X*_*i*_ by predicting the label of *f*_*i*_ with the learned binary classifier.

### Optimization

In this section, we derive approaches to solve the optimization problem constructed in equation (13). We first convert the constrained problem to an unconstrained problem by adding penalty functions. The resulting optimization problem becomes,


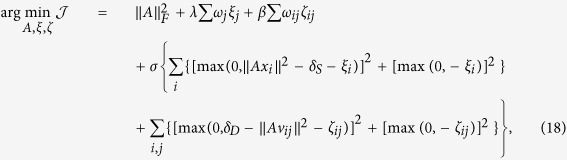


where *σ* is the penalty coefficient.

Then we use the gradient-projection method^36^ to solve the optimization problem in equation (18). To be precise, in the first step, we initialize *A*^0^, *ξ*^0^ and *ζ*^0^, and centralize the input data by equation (11). In the second step, we update the value of *A, ξ* and *ζ* using gradient descent based on the following rules,


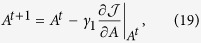



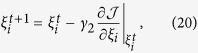



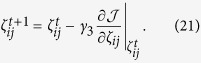


The derivatives of the objective *f* with respect to *A, ξ* and *ζ* in equations (19,20,21) are,


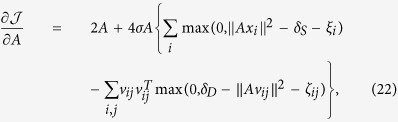



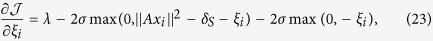



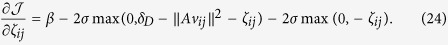


We repeat the second step until the change of the objective function 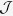 is less than a threshold *ε*. A detailed procedure is given in *Algorithm 1*.

## Results

In this section, we verify the effectiveness of the proposed MIMTL approach, by conducting extensive experiments on seven real-world organisms which cover the biological three-domain system^37^,^38^,^39^ (*i*.*e*., archaea, bacteria, and eukaryote). We compare the performance of MIMTL (The source code of MIMTL will be open upon the publication of papers.) with several sate-of-the-arts multi-instance learning methods including MIMLSVM^6^, MIMLNN^6^, EnMIMLNN^4^ and MICS^31^. And the results of the comparison show that the proposed algorithm MIMTL outperforms other algorithms.

### Data setting

The seven real-world organisms datasets (http://lamda.nju.edu.cn/files/MIMLprotein.zip) have been used by many prior researchers on genome-wide protein function prediction^4^ problem. The datasets come from the biological three-domain system (*i*.*e*., archaea, bacteria, eukaryote).

- **The Archaea Genomes**: Haloarcula marismortui (HM) and Pyrococcus furiosus (PF).
- **The Bacteria Genomes**: Azotobacter vinelandii (AV) and Geobacter sulfurreducens (GS).
- **The Eukaryote Genomes**: Caenorhabditis elegans (CE), Drosophila melanogaster (DM) and Saccharomyces cerevisiae (SC).

Details information of the datasets is shown in Tables 2 and 3. For each dataset, each bag containing several instances represents the protein in organisms, and each instance is described by a 216-dimensions vector where each dimension is the frequency of a triad type^40^. And a group of GO molecular function terms^41^ is associated with each instance. For example, the Haloarcula marismortui dataset contains 304 proteins (bags) and including a number of 234 gene ontology terms (class) on molecular function (Table 2). The total instance number of Haloarcula marismortui dataset is 950. The average number of instances per bag (protein) is 3.13 ± 1.09, and the average number of labels (GO terms) per instance is 3.25 ± 3.02.

For each dataset, we separate the bags into the source and target domain by a sampling procedure following MICS. For instance, we put the bag into source domain if 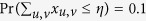 and 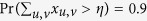 where 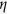 represents the median of 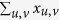
’s of all the bags in each dataset. The rest bags are put into the target domain. With this setting, the source domain has a higher density of bags with large feature values than that of the target domain.

Considering the fact that the segmentation methods of the source and target domains provided by MICS are artificially set. The data setting may be different from the real application. In order to give fair comparisons, we also test the performance of our algorithm in a more general setting. In this setting, the original bag data were randomly clustered into two clusters according to the center of the bag. Then, we randomly select one cluster as the source domain, and set the rest as the target domain. By this way, the original bag data is naturally split into two domains. Hence, the comparison will be more fair.

### Evaluation Measure

In our experiments, we use four popular evaluation criteria to evaluate the performance the multi-instance learning approaches, i.e., Ranking Loss (RankLoss)^6^, Coverage (Coverage)^42^, Average-Recall (avgRecall)^6^ and Average-F1 (avgF1)^6^. To explain each measure, for a given test set 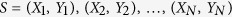, we denote *h*(*X*_*i*_) the returned labels for *X*_*i*_; *h*(*X*_*i*_, *y*) is the returned confidence (real-value) for *X*_*i*_; *rank*^*h*^(*X*_*i*_, *y*) is the rank of *y* which is derived from *h*(*X*_*i*_, *y*); 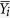 is the complementary set of *Y*_*i*_. Then, the criteria *Ranking Loss* is used to measure the average fraction of misordered label pairs generated by each algorithm. The little the ranking loss, the better the performance of the algorithm.


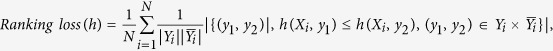


The criteria *Coverage* is utilized to evaluate the average fraction of how far it is needed to go down the list of labels to cover all of the proper labels in the test bag. The little the coverage, the better the performance of the algorithm.


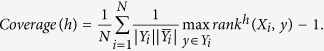


The criteria *Average*-*Recall* is included to measure the average fraction of correctly predicted labels. The larger the Average-Recall, the better the performance of the algorithm.


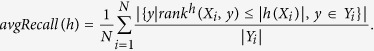


The criteria *Average*-*F1* is a tradeoff of the average precision^6^ and the average recall. The larger Average-F1, the better the performance of the algorithm.


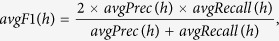


in which *avgPrec*(*h*) represents the average precision^6^.

Note that, in this paper, we do not use average precision to measure the performance of each algorithm. This is because, the positive and negative bags of these seven datasets are very unbalanced. The ratio between the number of positive instance and negative instance has been shown in Table 3. In this situation, if we set all the test bags to be negative, we can get a very high average precision. Hence, the average precision cannot measure the performance of each algorithm in the fair.

To make a fair comparison, we conduct all the experiments in this paper with 20 random permutations for each dataset. We report the comparison results for each evaluation metric-based on the averaging results over those 20 runs.

### Comparing Algorithms

In this section, we briefly introduce the comparison methods (MIMLNN, MIMLSVM, EnMIMLNN, MICS) (The codes of these four MIL algorithms have been shared by their authors: http://lamda.nju.edu.cn/CH.Data.ashx) used in our experiments. On one hand, considering the fact that MIMTL is used to tackle the multi-instance learning problem, we compare MIMTL with MIMLNN, MIMLSVM which are two classical multi-instance learning algorithms. On the other hand, since MIMTL is also a kind of metric-based MIL, we include EnMIMLNN as a comparison method. Considering the fact that, MIMTL and MICS are both designed for Multi-Instance learning under transfer learning setting. The difference between MIMTL and MICS is that these two methods use different distance to measure the bags’ distance. For instance, MIMTL use the Mahalanobis distance to measure the bags’ distance while MICS use the Euclidean distance. To verify the contribution of the Mahalanobis distance to MIMTL, we include MICS as a comparison method.

We have introduced two methods to predict bag label for the test bags. To research which method is better for MIMTL, we also compare MIMTL_*kNN*_ with MIMTL_*SVM*_ on all dataset. MIMTL_*kNN*_ is a variant of MIMTL by setting the base learner to be citation-kNN. And MIMTL_*kNN*_ is also a variant of MIMTL by setting the base learner to be SVM.

### Parameter Configurations

In this section, we present the detail parameter configurations of each algorithm used in our experiments. To make the comparison more fair, we use the best parameters reported in the papers for the baseline methods. We select the best parameters for MIMTL by cross-validation.

- *MIMLNN*: The regularization parameter used to compute the matrix inverse is set to 1 and the number of clusters is set to 40 percent of the training bags.
- *MIMLSVM*: The number of clusters is set to 20 percent of the training bags and the SVM used in MIMLSVM is implemented by LIBSVM^35^ package with radial basis function whose parameter “−c” is set to 1 and “−g” is set to 0.2.
- *EnMIMLNN*: The fraction parameter and the scaling factor are set to 0.1 and 0.8, respectively.
- *MICS*: The number of clusters is set to 80 percent of the training bags and the SVM used in MICS is implemented by LIBSVM^35^ package with radial basis function whose parameter “−*c*” is set to 1 and “−*g*” is set to 0.2.
- *MIMTL*: We set the balance parameters *λ* = 1, *β* = 1 and set the number of clusters to be 40 percent of the training bags. We select the radial basis function with “−*c* = 1” and “−*g* = 0.2” for the base learner SVM.

### Performance Comparison

**Figure 2:**
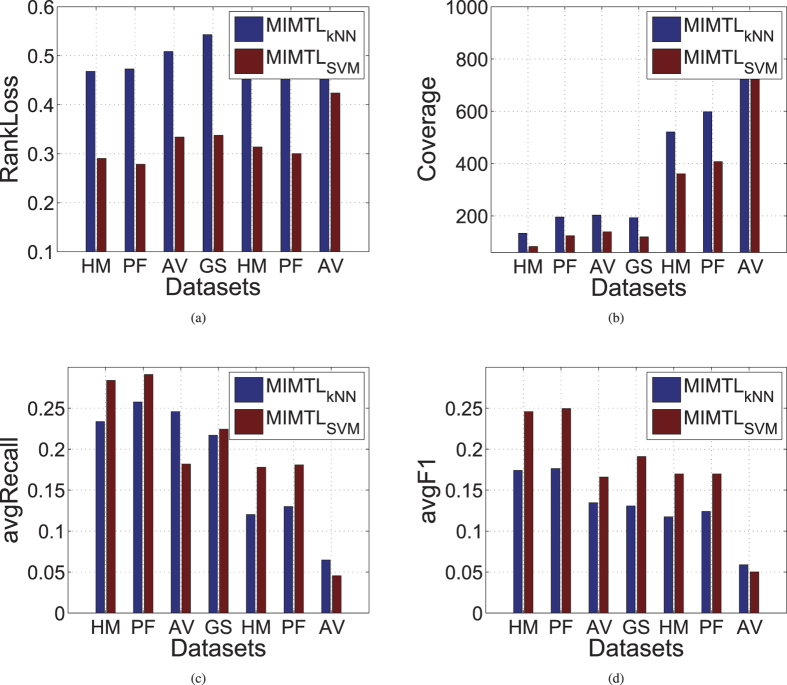
Comparison results with MIMTL*_k_NN* and MIMTL*_S_VM* on seven real-world organisms.

We have presented two versions of MIMTL, MIMTL_*kNN*_ and MIMTL_*SVM*_ in details. Before we compare MIMTL with other state-of-the-art MIL methods, we actually want to select a better base learner for MIMTL. To this end, we compare the performances of MIMTL_*kNN*_ and MIMTL_*SVM*_ on the seven datasets. Figure 2 reports the experimental results. From the figure, we can observe that the ranking loss and coverage of MIMTL_*SVM*_ on all the seven datasets is dramaticly lower than that of MIMTL_*kNN*_. We also note that the avgF1 and avgRecall of MIMTL_*SVM*_ on most datasets are much higher than that of MIMTL_*kNN*_. These experimental results in Fig. 2 suggest that SVM is more suitable for MIMTL as the base learner than citation-kNN.

In the second experiment, we verify the performance of MIMTL by comparing MIMTL with the other three traditional state-of-the-art MIL methods (MIMLNN, MIMLSVM, EnMIMLNN). The comparison results with four state-of-the-art MIL methods on seven real-world organisms are shown in Tables 4 and 5. From the table, we find that the performance of MIMTL is particularly significant than the other MIL methods. This is because, MIMLNN, MIMLSVM and EnMIMLNN are designed for the traditional MIL problem where the training and test bags are drawn from the same distribution. And the multi-instance classifier trained by MIMLNN, MIMLSVM and EnMIMLNN on SD cannot well suit to TD task. Different from MIMLNN, MIMLSVM and EnMIMLNN, MIMTL takes into account the distribution different problem between SD and TD and utilizes bag weights trick to handle this problem. Such that MIMTL can keep a better performance than other methods under transfer learning setting.

**Figure 3:**
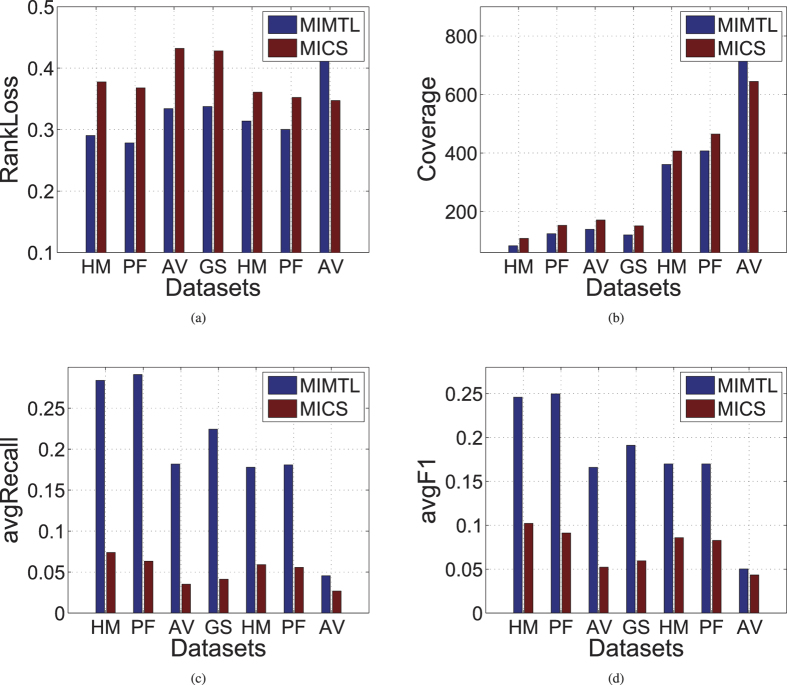
Comparison results with MIMTL and MICS on seven real-world organisms.

In the third experiment, we compare the performance of MIMTL with MICS since MIMTL is designed for multi-instance transfer learning problem, in this paragraph. The performance results for each algorithm on the seven datasets are shown in Fig. 3. From the figures, we find that the ranking loss and coverage of MIMTL on six of the seven datasets are lower than that of MICS. The avgRecall and avgF1 of MIMTL on all the seven datasets are higher than that of MICS. Though MIMTL and MICS are both designed for multi-instance transfer learning problem, the performance of MIMTL is much better than that of MICS. This may be because MICS only use the Euclidean distance to measure the distance bags. Compared to MICS, MIMTL utilize Mahalanobis distance into the multi-instance transfer learning. With the advantage of Mahalanobis distance, MIMTL can preserve much intrinsic geometric information of the bags than MICS. Hence, MIMTL can more effectively enhance the performance for multi-instance prediction for genome-wide protein function prediction.

In the fourth experiment, we also test the performance of MIMTL on the seven datasets with more general settings. In this experiment, we first randomly clustered each dataset in to two clusters. Then we set one cluster as the source domain and the rest as the target domain. Table 6 shows the experimental results. From the table, we can find that compared with four other baselines on the several genomic datasets, our algorithm is more excellent. Combining the experimental results from Tables 4 and 6, we found that our algorithm maintains excellent performance in both data settings (the data setting according to MICS, and the data setting according to random clustering). This may indicate that the performance of our algorithm is not affected by data settings. And this result also validates the robustness of our algorithm.

**Figure 4:**
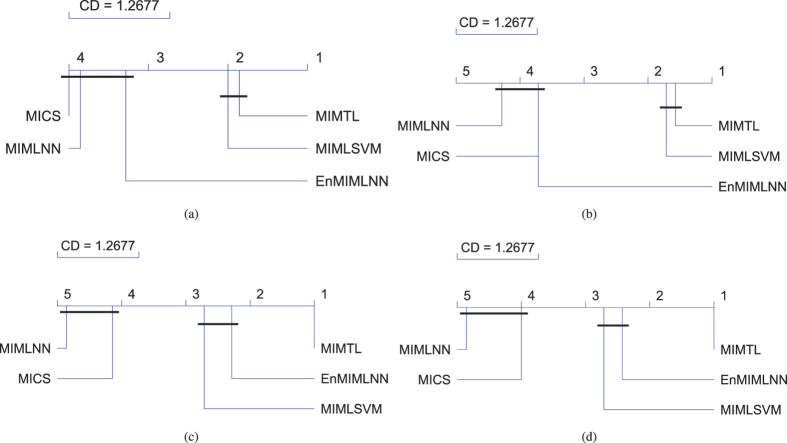
The average ranks diagrams^45^ for the ranking-based measures: Ranking Loss (**a**), Coverage (**b**), Average Recall (**c**), and Average-F1 (**d**). The data setting used in this figure is under the protocol of MICS^31^.

In the fifth experiment, we further verify compare MIMTL with the other state-of-the-art methods based on a robust non-parametric test (This non-parametric test provides us a method for comparing more algorithms across multiple data sets. The test procedure includes three steps: First, we rank the algorithms on each data set. Then, compute the average rank (in the descending order) of each algorithm on all the data set. At last, the Nemenyi post-hoc test is utilized to detect if a algorithm is significantly different from the others according to the average rank. The performances of two algorithms are significantly different if their corresponding average ranks differ by at least a critical distance (CD), vice versa. The algorithms that do not differ significantly than each other are usually connected with a bold horizontal line. The value of the critical distance is depended on the number of comparing algorithms, data sets number and a significance level *p (i*.*e*., *p* = 0.05). (The Friedman test^43^ with the corresponding Nemenyi post-hoc tests^44^) as recommended by Demšar, Janez^45^. The data setting used in this figure is following the protocol of MICS^31^. The test results of MIMLNN, MIMLSVM, EnMIMLNN, MICS and MIMTL are presented with several diagrams as shown in Fig. 4. Each subgraph in Fig. 4 is corresponding to a ranking-based measure. From the test results, we observe that the performance of MIMTL is similar as MIMLSVM on ranking loss and Coverage. And, the performance of MIMTL is significantly better than MIMLNN, EnMIMLNN, MICS on all the evaluation measures which verify the excellent performance of MIMTL.

Note that, the performance reported in Table 4 are different from that reported by EnMIMLNN and MIMLDML. This is because, we select the training and test data from different domains while EnMIMLNN select the training and test data from the same domain. To make the comparison more comprehensive, we compare MIMTL with the baselines following the evaluation protocol of EnMIMLNN, and randomly select the training and test data from the same domain.

## Additional Information

**How to cite this article:** Xu, Y. *et al*. Multi-Instance Metric Transfer Learning for Genome-Wide Protein Function Prediction. *Sci. Rep.* **7**, 41831; doi: 10.1038/srep41831 (2017).

**Publisher's note:** Springer Nature remains neutral with regard to jurisdictional claims in published maps and institutional affiliations.

## Figures and Tables

**Table 1:** Important Definitions.

Symbols	Definitions
SD	The source domain dataset.
TD	The target domain dataset.
*X*_*i*_	*i*-th bags, which represent a protein.
*x*_*j*_	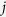 -th instance in a bag.
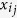	*j*-th instance in bag *X*_*i*_.
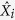	The average of all the instances in bag *X*_*i*_.
*Y*_*i*_	Represent the Gene Ontology terms assigned to *X*_*i*_.
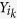	*k*-th element in *Y*_*i*_.
*c*_*i*_	The center of *X*_*i*_.
*D*(*x*_*i*_, *c*_*i*_)	The square of the Mahalanobis distance between instance *x*_*i*_ and *c*_*i*_.
*D*(*X*_*i*_, *X*_*j*_)	The square of the Mahalanobis distance between bags *X*_*i*_ and *X*_*j*_.
*A*	The learned Mahalanobis distance metric.
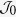	The loss corresponding to traditional Multi-Instance Metric Learning.
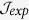	The expected loss.
*δ*_*S*_	A constant to limit the minimum distance between the center of the bag and the instance in the bag.
*δ*_*D*_	A constant to limit the maximum distance between bags from different class.
*ξ, ζ*	Two slack vectors to improve the robustness of the algorithm.
*ω*	The weight vector of bags.

**Table 2:** Characteristics of the seven datasets.

Genome	Bags	Classes	Instances	Dimensions	Bags in Source Domain	Bags in Target Domain
HM	304	234	950	216	152	152
PF	425	321	1317	216	213	212
AV	407	340	1251	216	204	203
GS	379	320	1214	216	190	189
CE	2512	940	8509	216	1256	1256
DM	2605	1035	9146	216	1303	1302
SC	3509	1566	6533	216	1755	1754

**Table 3:** Details information about positive and negative instances of the seven datasets.

	HM	PF	AV	GS	CE	DM	SC
Instances per bag	3.13	3.1	3.07	3.2	3.39	3.51	1.86
Labels per instance	3.25	4.48	4	3.14	6.07	6.02	5.89
Positives instance per classes	4.22	5.93	4.79	3.72	16.22	15.15	13.19
Positives instance/negative instance	1.41%	1.42%	1.19%	0.99%	0.65%	0.59%	0.38%

**Table 4:** Comparison results using the data setting of MICS^31^.

Genome	MIMLNN	MIMLSVM	EnMIMLmetric	MIMTL
Ranking Loss ↓				
HM	0.3411 ± 0.0229 (4)	0.3315 ± 0.0161 (3)	0.3248 ± 0.0092 (2)	0.2904 ± 0.0348 (1)
PF	0.3153 ± 0.0172 (3)	0.3443 ± 0.0149 (4)	0.3136 ± 0.0132 (2)	0.2784 ± 0.0197 (1)
AV	0.3887 ± 0.0167 (4)	0.3629 ± 0.0139 (2)	0.3882 ± 0.0049 (3)	0.3340 ± 0.0136 (1)
GS	0.4338 ± 0.0116 (4)	0.3979 ± 0.0153 (2)	0.4197 ± 0.0084 (3)	0.3376 ± 0.0224 (1)
CE	0.3929 ± 0.0138 (4)	0.2759 ± 0.0056 (1)	0.3643 ± 0.0103 (3)	0.3139 ± 0.0176 (2)
DM	0.3440 ± 0.0077 (3)	0.2797 ± 0.0056 (1)	0.3548 ± 0.0091 (4)	0.3002 ± 0.0145 (2)
SC	0.4081 ± 0.0071 (2)	0.2884 ± 0.0022 (1)	0.4083 ± 0.0067 (3)	0.4238 ± 0.0174 (4)
Average Rank	3.4286	2	2.8571	1.7143
Coverage ↓				
HM	104.9102 ± 6.1158 (4)	96.5986 ± 4.5074 (2)	99.1306 ± 3.6104 (3)	82.9551 ± 8.3830 (1)
PF	147.5855 ± 6.2129 (4)	147.3493 ± 7.0038 (3)	142.1638 ± 5.3214 (2)	124.2589 ± 7.3765 (1)
AV	168.0298 ± 5.7280 (4)	147.0354 ± 5.5012 (2)	160.9854 ± 2.6453 (3)	139.0677 ± 5.6253 (1)
GS	159.9543 ± 4.0170 (4)	139.6457 ± 4.5796 (2)	151.9766 ± 3.9327 (3)	120.0859 ± 8.4693 (1)
CE	453.8295 ± 13.6709 (4)	315.3995 ± 6.1653 (1)	421.4290 ± 9.1993 (3)	360.8714 ± 17.8819 (2)
DM	479.2888 ± 8.2933 (3)	376.5261 ± 7.0924 (1)	480.6691 ± 8.4298 (4)	407.3001 ± 18.8377 (2)
SC	834.1562 ± 11.0925 (3)	568.0499 ± 4.9514 (1)	834.9128 ± 9.2612 (4)	820.2915 ± 21.4581 (2)
Average Rank	3.7143	1.7143	3.1429	1.4286
Average-Recall ↑				
HM	0.0020 ± 0.0028 (4)	0.0951 ± 0.0185 (3)	0.1451 ± 0.0191 (2)	0.2840 ± 0.0377 (1)
PF	0.0039 ± 0.0031 (4)	0.0748 ± 0.0312 (3)	0.0859 ± 0.0086 (2)	0.2910 ± 0.0379 (1)
AV	0.0074 ± 0.0084 (4)	0.0548 ± 0.0132 (2)	0.0470 ± 0.0063 (3)	0.1819 ± 0.0221 (1)
GS	0.0055 ± 0.0053 (4)	0.0830 ± 0.0204 (3)	0.1405 ± 0.0343 (2)	0.2244 ± 0.0266 (1)
CE	0.0756 ± 0.0042 (4)	0.1022 ± 0.0045 (3)	0.1310 ± 0.0055 (2)	0.1779 ± 0.0163 (1)
DM	0.0499 ± 0.0058 (4)	0.0713 ± 0.0050 (3)	0.1104 ± 0.0067 (2)	0.1808 ± 0.0124 (1)
SC	0.0062 ± 0.0023 (4)	0.0289 ± 0.0010 (2)	0.0269 ± 0.0026 (3)	0.0455 ± 0.0235 (1)
Average Rank	4	2.7143	2.2857	1
Average-F1 ↑				
HM	0.0040 ± 0.0054 (4)	0.1315 ± 0.0220 (3)	0.2042 ± 0.0191 (2)	0.2459 ± 0.0433 (1)
PF	0.0076 ± 0.0060 (4)	0.1072 ± 0.0374 (3)	0.1342 ± 0.0103 (2)	0.2496 ± 0.0411 (1)
AV	0.0136 ± 0.0151 (4)	0.0800 ± 0.0173 (2)	0.0777 ± 0.0092 (3)	0.1659 ± 0.0245 (1)
GS	0.0107 ± 0.0099 (4)	0.1130 ± 0.0242 (3)	0.1775 ± 0.0325 (2)	0.1910 ± 0.0304 (1)
CE	0.1086 ± 0.0047 (4)	0.1418 ± 0.0046 (3)	0.1695 ± 0.0052 (2)	0.1698 ± 0.0129 (1)
DM	0.0781 ± 0.0076 (4)	0.1076 ± 0.0062 (3)	0.1458 ± 0.0064 (2)	0.1698 ± 0.0096 (1)
SC	0.0116 ± 0.0042 (4)	0.0473 ± 0.0014 (2)	0.0427 ± 0.0033 (3)	0.0502 ± 0.0194 (1)
Average Rank	4	2.7143	2.2857	1

↓ (↑) indicates the smaller (larger), the better of the performance.

**Table 5:** Comparison results using the evaluation protocol of EnMIMLNN^4^ (the source domain and target domain are drawn from the same distribution).

Genome	MIMLNN	MIMLSVM	EnMIMLNNmetric	MIMTL
Ranking Loss ↓				
HM	0.3146 ± 0.0218 (3)	0.3461 ± 0.0132 (4)	0.3096 ± 0.0236 (2)	0.2666 ± 0.0177 (1)
PF	0.3168 ± 0.0178 (2)	0.3557 ± 0.0138 (4)	0.3230 ± 0.0170 (3)	0.2859 ± 0.0159 (1)
AV	0.3721 ± 0.0159 (3)	0.3804 ± 0.0189 (4)	0.3707 ± 0.0127 (2)	0.3212 ± 0.0155 (1)
GS	0.3693 ± 0.0199 (2)	0.3813 ± 0.0250 (3)	0.3928 ± 0.0136 (4)	0.3194 ± 0.0192 (1)
CE	0.2307 ± 0.0033 (4)	0.1931 ± 0.0098 (1)	0.2097 ± 0.0061 (2)	0.2157 ± 0.0065 (3)
DM	0.2317 ± 0.0083 (4)	0.1893 ± 0.0049 (1)	0.2143 ± 0.0081 (3)	0.2126 ± 0.0098 (2)
SC	0.3090 ± 0.0066 (3)	0.2496 ± 0.0057 (1)	0.3352 ± 0.0073 (4)	0.2872 ± 0.0171 (2)
Average Rank	3	2.5714	2.8571	1.5714
Coverage ↓				
HM	102.5066 ± 5.1192 (3)	106.1454 ± 4.2328 (4)	99.5941 ± 4.6906 (2)	84.3914 ± 5.1049 (1)
PF	153.5061 ± 6.7742 (2)	158.7094 ± 5.0619 (4)	156.6540 ± 7.1480 (3)	137.2249 ± 7.1226 (1)
AV	168.5917 ± 6.1400 (4)	157.8088 ± 5.8091 (2)	157.9515 ± 5.5142 (3)	137.6652 ± 4.8167 (1)
GS	161.7774 ± 7.1352 (4)	149.8095 ± 7.5255 (2)	160.1811 ± 4.5556 (3)	127.2642 ± 6.4359 (1)
CE	317.1824 ± 4.2358 (4)	265.7309 ± 12.6447 (1)	287.8608 ± 6.8164 (3)	277.9165 ± 7.5019 (2)
DM	371.9936 ± 15.5735 (4)	307.6074 ± 8.2128 (1)	348.6276 ± 13.8078 (3)	318.3221 ± 19.4774 (2)
SC	726.7027 ± 13.0010 (3)	564.7905 ± 7.7727 (1)	754.1319 ± 11.8070 (4)	625.5630 ± 35.9015 (2)
Average Rank	3.4286	2.1429	3	1.4286
Average-Recall ↑				
HM	0.0633 ± 0.0081 (4)	0.1678 ± 0.0094 (3)	0.1803 ± 0.0174 (2)	0.3934 ± 0.0156 (1)
PF	0.0533 ± 0.0100 (4)	0.1264 ± 0.0136 (3)	0.1416 ± 0.0150 (2)	0.3632 ± 0.0259 (1)
AV	0.0546 ± 0.0116 (4)	0.1150 ± 0.0088 (3)	0.1279 ± 0.0134 (2)	0.2739 ± 0.0240 (1)
GS	0.0511 ± 0.0117 (4)	0.1286 ± 0.0129 (2)	0.1272 ± 0.0181 (3)	0.3092 ± 0.0180 (1)
CE	0.1671 ± 0.0079 (4)	0.2184 ± 0.0076 (3)	0.3170 ± 0.0132 (2)	0.5681 ± 0.0327 (1)
DM	0.1562 ± 0.0102 (4)	0.1926 ± 0.0088 (3)	0.2998 ± 0.0080 (2)	0.5710 ± 0.0289 (1)
SC	0.0350 ± 0.0028 (4)	0.0613 ± 0.0045 (3)	0.0739 ± 0.0055 (2)	0.4590 ± 0.0515 (1)
Average Rank	4	2.8571	2.1429	1
Average-F1 ↑				
HM	0.1073 ± 0.0114 (4)	0.2160 ± 0.0111 (3)	0.2465 ± 0.0166 (2)	0.3269 ± 0.0192 (1)
PF	0.0902 ± 0.0146 (4)	0.1684 ± 0.0138 (3)	0.1991 ± 0.0154 (2)	0.2965 ± 0.0212 (1)
AV	0.0898 ± 0.0151 (4)	0.1514 ± 0.0100 (3)	0.1770 ± 0.0159 (2)	0.2373 ± 0.0174 (1)
GS	0.0860 ± 0.0166 (4)	0.1647 ± 0.0151 (3)	0.1760 ± 0.0197 (2)	0.2549 ± 0.0130 (1)
CE	0.2306 ± 0.0083 (3)	0.2842 ± 0.0085 (2)	0.3808 ± 0.0113 (1)	0.1710 ± 0.0278 (4)
DM	0.2186 ± 0.0116 (3)	0.2577 ± 0.0098 (2)	0.3608 ± 0.0079 (1)	0.2013 ± 0.0255 (4)
SC	0.0587 ± 0.0040 (4)	0.0927 ± 0.0055 (3)	0.1065 ± 0.0061 (1)	0.1049 ± 0.0069 (2)
Average Rank	3.7143	2.7143	1.5714	2

↓ (↑) indicates the smaller (larger), the better of the performance.

**Table 6:** Comparison results on the dataset where the source and target domains are drawn from different clusters.

Genome	MIMLNN	MIMLSVM	EnMIMLmetric	MIMTL
Ranking Loss ↓				
HM	0.3281 ± 0.0279 (2)	0.3494 ± 0.0119 (4)	0.3333 ± 0.0276 (3)	0.3033 ± 0.0162 (1)
PF	0.3279 ± 0.0103 (2)	0.3524 ± 0.0039 (4)	0.3316 ± 0.0195 (3)	0.3035 ± 0.0231 (1)
AV	0.3786 ± 0.0110 (3)	0.3912 ± 0.0134 (4)	0.3772 ± 0.0155 (2)	0.3511 ± 0.0162 (1)
GS	0.3628 ± 0.0190 (2)	0.3722 ± 0.0098 (3)	0.3833 ± 0.0078 (4)	0.3353 ± 0.0226 (1)
CE	0.2304 ± 0.0029 (4)	0.1910 ± 0.0076 (1)	0.2099 ± 0.0044 (2)	0.2221 ± 0.0037 (3)
DM	0.2344 ± 0.0012 (4)	0.1892 ± 0.0012 (1)	0.2144 ± 0.0016 (3)	0.2057 ± 0.0082 (2)
SC	0.3062 ± 0.0056 (3)	0.2502 ± 0.0018 (1)	0.3371 ± 0.0071 (4)	0.2829 ± 0.0152 (2)
Average Rank	2.8571	2.5714	3	1.5714
Coverage ↓				
HM	105.4079 ± 2.7543 (4)	105.1294 ± 5.2652 (3)	103.8311 ± 5.4997 (2)	92.6952 ± 4.3571 (1)
PF	156.9030 ± 4.5401 (2)	157.1299 ± 3.8358 (3)	160.0532 ± 9.8530 (4)	135.1252 ± 10.3370 (1)
AV	167.2059 ± 9.4550 (4)	160.3971 ± 8.4329 (3)	156.1176 ± 6.9683 (2)	144.0719 ± 7.0694 (1)
GS	161.1491 ± 4.3131 (4)	147.8368 ± 5.6174 (2)	158.7298 ± 8.2310 (3)	134.2649 ± 10.7539 (1)
CE	316.9668 ± 6.1003 (4)	262.3989 ± 4.3975 (1)	286.4719 ± 2.7980 (3)	285.3007 ± 11.3299 (2)
DM	378.4963 ± 3.4863 (4)	311.0507 ± 7.4777 (1)	350.9775 ± 7.2447 (3)	312.6278 ± 12.7775 (2)
SC	718.7297 ± 8.7041 (3)	565.9772 ± 4.0773 (1)	754.0876 ± 17.5188 (4)	616.2332 ± 37.8974 (2)
Average Rank	3.5714	2	3	1.4286
Average-Recall ↑				
HM	0.0664 ± 0.0080 (4)	0.1713 ± 0.0102 (3)	0.1751 ± 0.0070 (2)	0.5435 ± 0.0456 (1)
PF	0.0525 ± 0.0054 (4)	0.1187 ± 0.0190 (3)	0.1423 ± 0.0124 (2)	0.6026 ± 0.0284 (1)
AV	0.0533 ± 0.0056 (4)	0.1088 ± 0.0072 (3)	0.1237 ± 0.0057 (2)	0.4727 ± 0.0176 (1)
GS	0.0455 ± 0.0060 (4)	0.1313 ± 0.0077 (2)	0.1158 ± 0.0192 (3)	0.4979 ± 0.0074 (1)
CE	0.1647 ± 0.0050 (4)	0.2205 ± 0.0094 (3)	0.3090 ± 0.0092 (2)	0.6066 ± 0.0186 (1)
DM	0.1590 ± 0.0119 (4)	0.1920 ± 0.0085 (3)	0.3036 ± 0.0081 (2)	0.5785 ± 0.0279 (1)
SC	0.0319 ± 0.0020 (4)	0.0610 ± 0.0038 (3)	0.0769 ± 0.0043 (2)	0.4752 ± 0.0483 (1)
Average Rank	4	2.8571	2.1429	1
Average-F1 ↑				
HM	0.1121 ± 0.0116 (4)	0.2209 ± 0.0125 (3)	0.2387 ± 0.0060 (2)	0.2814 ± 0.0528 (1)
PF	0.0895 ± 0.0079 (4)	0.1600 ± 0.0202 (2)	0.1994 ± 0.0146 (1)	0.1595 ± 0.0137 (3)
AV	0.0877 ± 0.0074 (4)	0.1414 ± 0.0069 (3)	0.1699 ± 0.0037 (1)	0.1599 ± 0.0111 (2)
GS	0.0786 ± 0.0089 (4)	0.1700 ± 0.0092 (2)	0.1660 ± 0.0226 (3)	0.1846 ± 0.0481 (1)
CE	0.2280 ± 0.0046 (3)	0.2871 ± 0.0102 (2)	0.3739 ± 0.0057 (1)	0.1584 ± 0.0150 (4)
DM	0.2226 ± 0.0124 (3)	0.2570 ± 0.0095 (2)	0.3629 ± 0.0075 (1)	0.2037 ± 0.0320 (4)
SC	0.0543 ± 0.0031 (4)	0.0922 ± 0.0047 (3)	0.1097 ± 0.0054 (1)	0.1001 ± 0.0142 (2)
Average Rank	3.7143	2.4286	1.4286	2.4286

↓ (↑) indicates the smaller (larger), the better of the performance.
